# Design, Calibration, and Application of a Robust, Cost-Effective, and High-Resolution Lensless Holographic Microscope

**DOI:** 10.3390/s22020553

**Published:** 2022-01-11

**Authors:** Jose Angel Picazo-Bueno, Karina Trindade, Martin Sanz, Vicente Micó

**Affiliations:** Optics and Optometry and Vision Science, University of Valencia, 46100 Burjassot, Spain; karinri@alumni.uv.es (K.T.); martin.sanz@uv.es (M.S.); vicente.mico@uv.es (V.M.)

**Keywords:** in-line holographic microscopy, digital holography, Gabor holography, lensless microscope, digital image processing, medical and biological imaging, microscope characterization

## Abstract

Lensless holographic microscope (LHM) is an emerging very promising technology that provides high-quality imaging and analysis of biological samples without utilizing any lens for imaging. Due to its small size and reduced price, LHM can be a very useful tool for the point-of-care diagnosis of diseases, sperm assessment, or microfluidics, among others, not only employed in advanced laboratories but also in poor and/or remote areas. Recently, several LHMs have been reported in the literature. However, complete characterization of their optical parameters remains not much presented yet. Hence, we present a complete analysis of the performance of a compact, reduced cost, and high-resolution LHM. In particular, optical parameters such as lateral and axial resolutions, lateral magnification, and field of view are discussed into detail, comparing the experimental results with the expected theoretical values for different layout configurations. We use high-resolution amplitude and phase test targets and several microbeads to characterize the proposed microscope. This characterization is used to define a balanced and matched setup showing a good compromise between the involved parameters. Finally, such a microscope is utilized for visualization of static, as well as dynamic biosamples.

## 1. Introduction

Light microscopy is one of the most employed techniques for analysis in biomedicine, mainly due to its capability to provide images of microscopic specimens using a real-time and non-invasive operation principle. In that technique, high-quality imaging is typically provided by optical compound microscopes. However, such microscopes are relatively bulky, and often include complicated lens systems which can significantly increase their prices. Those aspects can negatively restrict their use only to advanced laboratories and/or make them neither field-portable nor affordable in practice for developing countries [1]. By contrast, lens free devices are ideal for field applications since they offer small-size and light-weight digital imaging portable platforms [2,3]. In particular, lensless holographic microscopes were reported in the past years, providing a successful miniaturized solution for, just to cite some examples, global healthcare monitoring, point-of-care diagnosis, and biomedical applications [4]. As a common point, lensless microscopes are based on cost-effective, light-weight, compact, and portable imaging devices that greatly improve rapid and accurate diagnosis in the field-setting [5,6,7,8,9,10,11,12,13,14,15,16,17,18,19,20,21,22].

Lensless holographic microscopy (LHM) arises from the digital implementation of Gabor holography [23,24]. In LHM, an extremely simple layout is assembled using very simple and few components. A coherent point light source illuminates a sample under analysis. Then, the light scattered by the sample interferes with the light coming directly from the point source at the sensor area, recording a digital Gabor hologram [25]. Finally, imaging is performed by using numerical diffraction equations and digital image processing tools [26]. Different ways to obtain the spherical point source were reported in LHM [5,6,7,15,25,27,28,29,30,31,32], such as the use of a pinhole in combination with a laser beam [6,25,27], a LED and objective lens [5], or just with a LED [7], or by illumination through GRIN lenses [14,28], fiber optics [29], a pulsed laser radiation [30], a SLED source [31], a terahertz laser [33], a laser diode [15], or a laser diode in combination with a tunable lens [32].

LHM is usually implemented using two opposite arrangements with particular significance, depending on the position of the sample between the illumination point source and the digital sensor. When the sample is located in close proximity to the illumination source but far from the digital sensor plane, the microscope implements a digital in-line holographic microscopy (DIHM) configuration [6,25,27]. In DIHM, the hologram is geometrically projected with magnification values ranging from 5X to 20X. In that case, retrieved images usually present similar features to the provided by conventional digital holographic microscopy concerning magnification, field of view (*FOV*), and resolution when using objectives of medium numerical apertures (NAs) (~0.4–0.5 NA) [34], although higher resolution images were also presented [35,36,37]. On the opposite case, commonly known as on-chip microscopy, the sample is positioned just ahead of the digital sensor, whereas the illumination source remains far from it [7,8,9]. On-chip microscopy is characterized by not inducing magnification (~1X) by geometry, meaning that the *FOV* is practically equal to the size of the sensitive area of the digital sensor. By contrast, the resolution is usually modest incoming from relatively low *NA* values (~0.2 *NA*) and limited by the pixel geometrical constraints, and not by diffraction unlike DIHM. Nonetheless, several pixel superresolution approaches were also implemented in order to decrease the effective pixel size [29,38,39]. Finally, other intermediate cases, in addition to such opposite layouts, were also reported in the literature [18,40]. 

Due to its strong practical potential and applicability, LHMs have been mainly proposed under an application-oriented perspective, that is, validation of their capabilities to monitor/measure/analyze/quantify/etc. different items (from biomedical properties to advanced statistical parameters) but aimed at specific applications. However, theoretical–practical analysis and characterization of the performance in a LHM is not as common in the literature where only a few references partially cover these issues [6,21]. For that reason, we present in this contribution a complete analysis of the performance of a compact, reduced cost, and high-resolution lensless holographic microscope based on DIHM for biomedical applications. Thus, different optical parameters such as lateral resolution, lateral magnification, *FOV*, and extended depth of field (DOF) are interrelated and discussed into detail, comparing the experimental results with the expected theoretical predictions for different axial positions of the sample. Moreover, we utilize the microscope to characterize static as well as dynamic biosamples. Hence, Section 2 includes a description of our microscope, mathematical foundations, and sample preparations. In Section 3, we present the experimental results achieved, first in the calibration process, and second in the application of the lensless microscope to the study of undyed biosamples. Finally, Section 4 covers the discussion and main conclusions of the contribution.

## 2. Materials and Methods

### 2.1. Description of the Lensless Holographic Microscope

**Figure 1:**
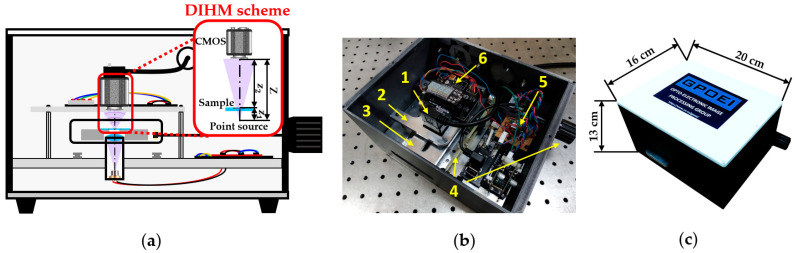
Presentation of the lensless holographic microscope. (**a**) Assembly diagram of a central cross-section of the microscope including the DIHM scheme. (**b**) Perspective view of the microscope once the top cover is taken off. (**c**) Global view of the microscope with external dimensions.

Our lensless microscope is presented in Figure 1 by means of a diagram and two general pictures. Figure 1a shows an assembly diagram of a central cross-section of the microscopes, including the DIHM scheme, in order to visualize different components and trace the light path inside the microscope. A low-cost laser diode source taken from a Blu-ray optical unit emits light at 405 nm diverging towards a high *NA* focusing lens. The light emerging from such a lens is focused on a point located less than 1 mm away from the lens, creating a quasi-point light source. In order to define a DIHM configuration, the sample is placed in close proximity to such a spherical divergent illumination source at a distance *z*_1_. The interference pattern generated by the coherent overlapping between the light scattered and not scattered by the object is recorded by a monochrome digital sensor (Ximea XiQ USB3, sensor CMOSIS CMV4000, model MQ042MG-CM, 2048 × 2048 pixels, 5.5 μm pixel size, 90 fps full frame), thus creating an in-line digital hologram at a distance *Z* away from the divergent illumination. 

In the picture included in Figure 1b, the microscope appears without the top cover, so that we can see the different mechanic and electronic components integrating the device. The microscope is designed with a commercially available CAD software platform and built employing Fused Deposition Material with ABS for the external structure and a mechanization process to assembly the internal elements. Inside the microscope, we can distinguish: (1) a CMOS camera with its USB connector; (2) a mechanical stage that holds the camera; (3) a metallic platform that supports the sample, having a slot centered to the optical axis of the microscope in order to leave the light passing through it; (4) an external knob and internal transmission system to extract and longitudinally move the sample stage; (5) an electronic circuit that controls the laser diode emission power; and (6) an automatic temperature controller (power supply system and LED display) connected to the sample stage with the aim of keeping living biosamples at proper temperature. Notice that the laser diode and the focusing lens are hidden by the sample stage. All those mechanical, optical, and electronic components are in a compact way inside the microscope, having external dimensions of 20 cm × 16 cm × 13 cm (see Figure 1c).

### 2.2. Mathematical Foundations

#### 2.2.1. Recording and Reconstruction Processes

The mathematical principles of our lensless holographic microscope are based on DIHM. In DIHM, the object is illuminated with a spherical divergent beam. Part of this light is scattered by the object, considered as object beam, whereas the other part is not, forming the reference beam. Then, both beams interfere at the recording plane, creating an in-line digital hologram. Assuming that the complex amplitude of the object and reference beams are $A_{obj}\left(x,y\right)$ and $A_{ref}\left(x,y\right)$ at the recording plane, respectively, being (*x*,*y*) the spatial coordinates at the recording plane, then the intensity distribution in such a plane is given by,

(1) $$\begin{array}{l} I\left(x,y\right)={\left|A_{obj}\left(x,y\right)+A_{ref}\left(x,y\right)\right|}^{2}\\ \;\;\;={\left|A_{obj}\left(x,y\right)\right|}^{2}+{\left|A_{ref}\left(x,y\right)\right|}^{2}+A_{obj}\left(x,y\right)\cdot A_{ref}^{*}\left(x,y\right)+A_{obj}^{*}\left(x,y\right)\cdot A_{ref}\left(x,y\right) \end{array}$$

being * the complex conjugate of the complex amplitude. The first two terms on the right side in Equation (1) are the intensities coming from the object and reference beams, respectively. On the other hand, the last two terms are the twin images charateristic of classical holography, which are produced symmetrically with respect to the hologram at the same distance. 

The complex amplitude distribution at the object plane can be reconstructed by digital propagation of the hologram using scalar diffraction theory [41]. However, such a complex amplitude appears disturbed by the superposition of the other terms also present in the hologram. Nonetheless, the efficacy of the reconstruction process increases when $A_{ref}\left(x,y\right)\gg A_{obj}\left(x,y\right)$, that is, when the light scattered by the object is small in comparison to the unscattered light. In such a case, the complex amplitude distribution at the object plane is only affected by the presence of a defocused diffraction pattern produced by the twin image, since the first term in Equation (1) can be neglected ${\left|A_{obj}\left(x,y\right)\right|}^{2}\ll A_{obj}\left(x,y\right)\cdot A_{ref}^{*}\left(x,y\right)$, and the second term ${\left|A_{ref}\left(x,y\right)\right|}^{2}$ is just the recording of a spherical wave whose intensity covers all sensor area, which only causes a slow and radial decreasing of the background intensity. In our case, we employ numerical diffraction based on angular spectrum method (ASM) [41] to propagate the hologram backwards until the object plane by using the following expression,

(2) $$E\left(x_{o},y_{o};z_{1}\right)=F^{-1}\left\{F\left\{\sqrt{I\left(x,y\right)}\right\}\left[k_{x},k_{y}\right]\cdot e^{ik_{z_{2}}z_{2}}\cdot circ\left(\frac{\sqrt{k_{x}^{2}+k_{y}^{2}}}{k}\right)\right\}\left[x_{o},y_{o}\right]$$

In Equation (2), F{} and $F^{-1}\{\}$ are the two-dimensional (2D) Fourier and inverse Fourier transforms, respectively, z_2_ is the distance between the recording and object planes (see Figure 1a), $k_{x}$ and $k_{y}$ are the spatial frequencies, *k* is the wavenumber k=2π/λ, $k_{z_{2}}=\sqrt{k^{2}-k_{x}^{2}-k_{y}^{2}}$, and circ() is the circle function, whose value is one where the argument is less than one ($k^{2}\geq k_{x}^{2}+k_{y}^{2}$) and is zero otherwise. Finally, $E\left(x_{o},y_{o};z_{1}\right)$ is a complex distribution which contains, among other things previously indicated, the amplitude and phase distribution of the light field at the object plane. Owing to the ASM being based on propagation of plane waves, it does not present any distance *z*_2_ limitation [41].

#### 2.2.2. Optical Parameters

All optical microscopes are partly characterized by a set of different optical parameters that give an idea of the aspect and quality of the images provided by them. Perhaps the most significant parameters when describing a microscope are lateral magnification, *FOV*, NA, and lateral and axial spatial resolutions. In the following, we introduce the mathematical expressions that define such parameters for a lensless microscope. 

In DIHM, the lateral magnification *M* comes from the geometric projection of the sample from the point source onto the recording plane. Hence, *M* only depends on the ratio between the axial distances *z*_1_ and *Z* according to the expression [5],

(3) $$M=\;\frac{Z}{z_{1}}$$

By contrast, the *FOV* is directly related to *M* and the sensor area of the digital camera, and can be calculated as

(4) $$FOV=\;{\left(\frac{Np}{M}\right)}^{2}$$

*N* being the number of pixels and *p* being the pixel size of the camera.

Regarding the lateral resolution *ρ* in DIHM, it can be limited by several factors, such as diffraction, spatial aliasing, limited spatial and temporal coherence of the source, or reduced signal-to-noise ratio (SNR) [42]. Here, we will consider that the diffraction and aliasing are predominant factors in the restriction of the resolution of the microscope, instead of the coherence of the light source or the SNR. Hence, the resolution limit imposed by diffraction theory is based on the relation between the wavelength *λ* of the illumination source and the *NA* of the imaging system in the way,

(5) $$\rho_{diff}=k\frac{\lambda }{NA}$$

where *k* is a constant parameter that takes different values for incoherent (*k* = 0.61) and coherent (*k* = 0.82) light, and for near-field imaging (*k* = 1), all of them given for circular apertures and following the Rayleigh criterion [43]. DIHM utilizes diffraction in the Fresnel regime, since normally the Fresnel number in that configuration is $F=a^{2}/z\lambda \geq 1$, *a* being the radius of the circular aperture, and *z* being the distance from such an aperture. For our specific case, where *z*~15 mm, *λ* = 405 nm, and *a*~6 mm, the Fresnel number is *F*~6000 >> 1. Hence, we will consider that *k* = 1 in our case, despite being our imaging system limited by the sensor area (square aperture). Additionally, *NA* is directly related to half of the size of the sensor area (*Np*/2) and its distance *z*_2_ with the object plane as [43],

(6) $$NA=\sin\left(\tan^{-1}\left(\frac{Np}{2z_{2}}\right)\right)\;$$

On the other hand, the geometrical parameters of the digital sensor can also reduce the resolution achieved by the lensless microscope, according to the Nyquist-Shannon sampling theorem [44]. Thus, any detail of the object smaller than a size given by the following equation,

(7) $$\rho_{geo}=\frac{2\cdot p}{M}\;$$

cannot be well sampled by our digital system, so that the smallest elements will be lost in the digital recording process.

In addition, the axial resolution is inversely proportional to the squared *NA* and is given by the following expression

(8) $$\rho_{axial}=\frac{\lambda }{NA^{2}}$$

Finally, the proposed lensless holographic microscope also allows phase imaging. Numerical propagation of a digital hologram provides a complex amplitude distribution, containing not only intensity but also phase information of the light field in a given plane. Thus, if we propagate towards the image plane of a phase object, we can achieve the phase delays introduced by the different elements of that object, which is directly related to the thickness and the refractive index (RI) of such elements, according to the expression,

(9) $$\Delta \phi \left(x,y\right)=\frac{2\pi }{\lambda }\left(n_{s}\left(x,y\right)-n_{m}\left(x,y\right)\right)\cdot t\left(x,y\right)$$

being $n_{s}\left(x,y\right)$ and $n_{m}\left(x,y\right)$ the RI of the sample and the surrounding medium, respectively, and t(x,y) the thickness distribution of the sample. In the particular case of having a sample with a homogeneous medium, $n_{m}\left(x,y\right)=n_{m}=cte$. 

### 2.3. Sample Preparation

Three different samples, namely, synthetic microspheres, fixed prostate cancer cells, and live sperm cells, are prepared following different procedures. On one hand, the polystyrene microbeads with 3 μm in diameter are first homogenized, and second placed in an Eppendorf tube, where they are highly diluted into water (1:100). After that, we pipette one drop of the solution on a microscope slide and another one on a cover glass. Once the water is evaporated and the samples are completely dry, we build a lab-made chamber where both surfaces containing microspheres are facing each other and separated by 1 mm.

On the other hand, two different types of prostate cancer cells (PC-3 and LnCaP cell lines) are prepared following a similar procedure. The cells are cultured in a RPMI 1640 medium with 10% fetal bovine serum, 100 U/mL Penicillin, and 0.1 μg/mL streptomycine at standard cell culture conditions (37 °C) in 5% CO_2_ in a humidified incubator. Once the cells reach a confluent stage, they are released from the culture support and centrifuged. The supernatant fluid is discarded by centrifugation, and the cells are resuspended in a cytopreservative solution and mounted in a microscopy slide.

Finally, a human commercial insemination dose from Proiser R+D S.L. is received after conventional refrigerated transport (less than 24 h). After homogenization of the dose, 1 mL is placed in an Eppendorf tube, maintained for 20 min at 37 °C. Next, the semen sample is inserted by pipetting into a commercially available counting chamber (100 μm thickness). 

## 3. Results

### 3.1. Calibration of the Lensless Holographic Microscope

Our lensless microscope is first calibrated using a positive high-resolution USAF test target (Model HIGHRES-2, Newport, 137 nm minimum bar width (G11-E6)). The target area is a quartz substrate with opaque lines of chrome with 100 nm in thickness. In this process, we obtain the calibration curves concerning lateral magnification (Section 3.1.1), *FOV* (Section 3.1.2), lateral resolution (Section 3.1.3), and axial resolution (Section 3.1.4) as a function of the axial distance *z*_1_ between the point source and the sample, while keeping fixed the distance Z between both source and recording planes. Here, the experimental results are compared to the theoretical values computed when considering the corresponding expressions of the Section 2.2. In addition, the imaging system is analyzed in terms of depth of field (DOF) (Section 3.1.5) and phase imaging (Section 3.1.6) using a 3 μm microspheres sample and a quantitative phase target, respectively.

**Figure 2:**
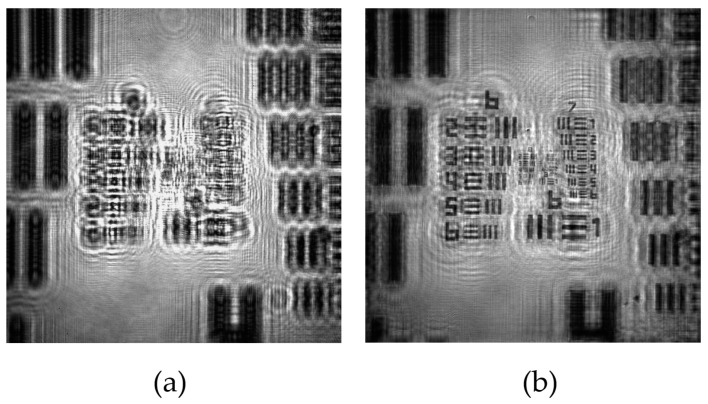
Example of the recording and reconstruction images of our lensless holographic microscope for the case of a positive USAF test target. (**a**) Recorded digital in-line hologram; (**b**) Retrieved intensity image provided by ASM.

The calibration process is carried out by means of the analysis of the retrieved images after numerical refocusing by ASM propagation of the recorded in-line hologram (Figure 2a) towards the image plane (Figure 2b). For the obtention of the calibration curves of the magnification, *FOV*, and resolutions, we perform the experimental analysis considering 9 different distances *z*_1_ within the interval 0.5 ± 0.1 mm to 5.0 ± 0.1 mm. *z*_1_ is controlled by placing the sample onto a manual XYZ linear translation stage, having an axial accuracy of 0.1 mm. Note that those distances are small in comparison to the distance Z, whose value is *Z* = 15.0 ± 0.1 mm, so that a DIHM configuration is defined for all distances considered here. 

#### 3.1.1. Lateral Magnification

**Figure 3:**
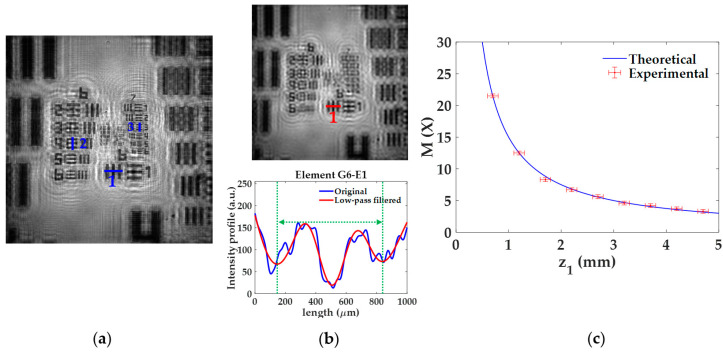
Experimental procedure for the analysis of the lateral magnification *M*. (**a**) Retrieved intensity image for a distance *z*_1_ = 0.7 ± 0.1 mm; (**b**) low-pass filtered image of (**a**) (upper part) and comparison between intensity profiles for better obtention of *M* values (bottom part); (**c**) calibration curve of *M* with the distance *z*_1_ including experimental and theoretical values.

The lateral magnification M provided by the lensless microscope is quantified for different *z*_1_ distances of the USAF test target. The experimental procedure considering *z*_1_ = 0.7 ± 0.1 mm and the final calibration curve are included in Figure 3. In the experimental procedure, for a given *z*_1_, we retrieved the image of the USAF test target and compute M considering three elements of different groups located at different regions of the image. For that, we measure the spatial period of the vertical/horizontal bars of such elements by plotting the intensity profiles along the blue lines included in Figure 3a. Since the retrieved image provides a noisy profile, where neither the borders nor the center of the bars appears well-defined, we apply a low-pass filter to the image (see upper part in Figure 3b), thus removing the high frequency noise and having a more periodic intensity profile from which the spatial period can be computed (see lower part in Figure 3b). After that, we measure the distance between two local minima (see green dashed lines included in Figure 3b) and divide by 2 to obtain the spatial period. This is performed to reduce half the experimental error. The local minima are associated with the center of the bars, thus providing a realiable period of the element. For the case depicted in Figure 3, we consider the elements G6-E1 (line 1), G6-E4 (line 2), and G7-E3 (line 3), whose spatial periods are 15.63, 11.05, and 6.20 μm, respectively. When measuring such periods in the image, they extend 339 ± 3 μm, 234 ± 3 μm, and 133 ± 3 μm, which correspond to lateral magnifications of 21.7 ± 0.2X, 21.2 ± 0.3X, and 21.5 ± 0.5X, respectively. Finally, the weighted average of the previous values provides a final magnification value of M = 21.5 ± 0.3X.

Once the previous process is performed for the 9 different *z*_1_ distances, we finally obtain the experimental magnification values, marked with red circles and error bars in Figure 3c. Figure 3c also includes the theoretical values of the magnification (blue line) provided by Equation (3) for such *z*_1_ distances. Looking at Figure 3c, we can state that the experimental values are in good agreement with the theoretical ones. As expected by analyzing Equation (3), the magnification increases quickly when the object is approaching to the illumination point source, whereas its variation is not so remarkable in the other case. For instance, if the sample is displaced from an initial position of *z*_1_ = 1 mm towards a final position of *z*_1_ = 0.5 mm, the magnification grows rapidly from 15× to 30×. By contrast, when considering the same change of 0.5 mm between *z*_1_ = 4.5 mm and 5 mm, the magnification only ranges from 3.3× to 3×. 

#### 3.1.2. Field of View

**Figure 4:**
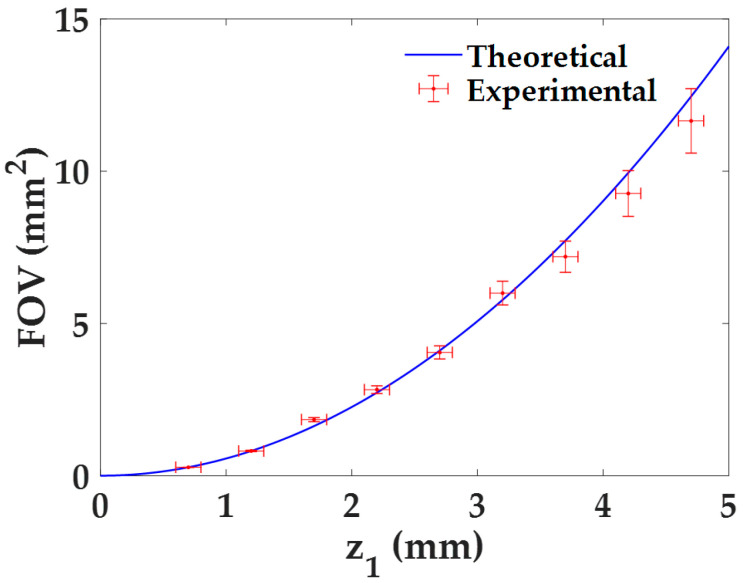
*FOV* characterization as a function of the distance *z*_1_ for the proposed lensless holographic microscope.

The quantification of the *FOV* with the distance *z*_1_ is directly derived from the previous analysis for the lateral magnification by considering Equation (4). Indeed, both the experimental results and the theoretical values are directly derived from it, whose experimental errors are calculated using the error propagation formula, $\delta_{FOV}=$ $\left|-\frac{{\left(Np\right)}^{2}}{M^{3}}\right|\cdot \delta_{M}$, being *δ_M_* the experimental error of the magnification *M*. The dependence of the *FOV* with the distance *z*_1_ is presented in Figure 4. As we can see, the *FOV* increases quadratically with the distance *z*_1_, approaching to 0 mm^2^ when *z*_1_ → 0 mm. This behavior can be also seen when combining Equations (3) and (4). Furthermore, the experimental values perfectly match with the theoretical ones.

#### 3.1.3. Lateral Resolution

For the analysis of the lateral resolution *ρ* of the microscope as a function of *z*_1_, we perform the following procedure: (1) we visualize which elements of the test target are close to the resolution limit at best focused image; (2) we perform an intensity profile along the perpendicular direction of such elements; (3) we quantify the intensity differences between the chromed elements, their immediate background, and the external background; and (4) when the central peaks (immediate background) are found to rise about or more than 27% [44] above the dip intensity (chromed element) with respect to the external background, then the element is considered as resolved.

**Figure 5:**
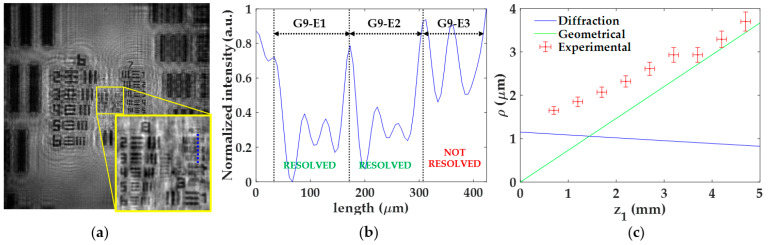
Experimental procedure for the analysis of the lateral resolution *ρ*. (**a**) Focused image for the distance *z*_1_ = 0.7 ± 0.1 mm; (**b**) intensity profile along blue line included in (**a**) for resolution limit determination; (**c**) calibration curve of the lateral resolution considering both experimental values and theoretical resolution limits according to diffraction and geometrical constraints.

Figure 5 includes the experimental procedure considering *z*_1_ = 0.7 ± 0.1 mm as well as the calibration curve. Here, the best focused image is shown in Figure 5a, where the region enclosed in the yellow square is magnified for better visualization of the elements closer to the resolution limit. An intensity profile of such elements (blue dashed line in Figure 5a) is presented in Figure 5b, where the different elements are separated by black dashed lines. After calculating the previously mentioned intensity differences, we can state that the elements G9-E1 and G9-E2 are well-resolved (we can identify the three chromed bars of those elements), whereas G9-E3 remains unresolved. Hence, the lateral resolution provided by the microscope for this *z*_1_ is a value between 1.74 μm (G9-E2 resolved) and 1.55 μm (G9-E3 not resolved). The final resolution limit considered here is the intermediate resolution value (*ρ*_exp_ = 1.65 ± 0.09), whereas the uncertainty in the measurement is the difference between such values.

Once the procedure is performed for all *z*_1_, we then represent in a single plot (Figure 5c) the lateral resolution experimentally obtained (red circles with error bars) and the theoretical resolution limits calculated from Equations (5) and (7), according to diffraction (blue line) and geometrical pixel constraints (green line), respectively. Looking at the theoretical resolution limits in Figure 5c, we can notice that the profiles cross each other at *z*_1_ = 1.45 mm. In addition, we can see that the resolution is limited by diffraction for *z*_1_ values smaller than the crossing point while, for higher values, the geometrical constraints of the digital sensor begin to reduce the lateral resolution. Furthermore, the resolution limit is approximately stable around 1.1 μm below the crossing point, whereas a progressive loss of resolution is obtained when *z*_1_ is above of it and ranging from 1.1 μm (*z*_1_ = 1.45 mm) to 3.7 μm (*z*_1_ = 5 mm). By contrast, the experimental resolution limit is progressively increasing from 1.65 ± 0.09 μm to 3.7 ± 0.2 μm when changing from *z*_1_ = 0.7 ± 0.1 mm to *z*_1_ = 4.7 ± 0.1 mm. Moreover, those experimental values are above these theoretical ones and sometimes differ significantly from them. A reason for such a discrepancy could be because the test target utilized for the resolution analysis does not fully satisfy the Gabor’s condition. Since the USAF test target presents different chromed elements of different sizes, it could be that some of them block a considerable part of the reference light necessary to have interferences of high frequency, thus reducing the number of interference fringes employed for reconstruction and eventually the resolution. In addition, the presence of the twin image may also hinder the resolution analysis. In any case, and leaving aside the discrepancy between theoretical and experimental values, the experimental trend has a reasonable behavior since it approaches the geometrical resolution limit as *z*_1_ increases. 

#### 3.1.4. Axial Resolution

**Figure 6:**
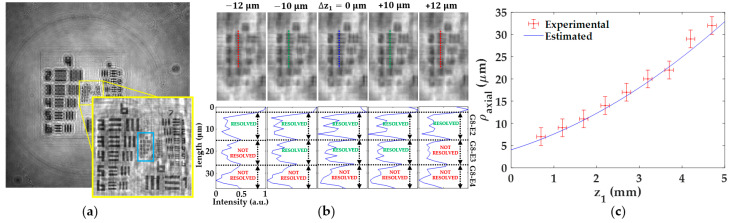
Analysis of the axial resolution of the lensless holographic microscope. (**a**) Best focused image for the case of *z*_1_ = 3.2 ± 0.1 mm. (**b**) Propagated images (upper part) of the region enclosed in the blue rectangle included in (**a**) and its corresponding intensity profiles along the dashed lines. (**c**) Calibration curve of the axial resolution including both experimental and expected values.

The axial resolution of our lensless microscope is also experimentally analyzed with respect to the distance *z*_1_. For that purpose, we first find the best focused image for each *z*_1_, setting this axial position as Δ*z*_1_ = 0 ± 2 μm, where 2 μm is the axial digital step between propagated planes considered here. Next, we digitally propagate such focused image backward and forward until the test target element defining the lateral resultion limit will be lost. Here, we use the same resolution criterion as in Section 3.1.3. Finally, the axial resolution of the microscope for that distance *z*_1_ is taken as the maximum numerical propagation distance that still allows to resolve the test element defining the resolution limit at the distance *z*_1_. Figure 6 includes the experimental procedure for a distance *z*_1_ = 3.2 ± 0.1 mm (see Figure 6a,b). Figure 6a presents the best focused image, including a magnified image of the central area clearly showing the lateral resolution limit (G8-E3 as last resolved element). This best focused image is propagated along an axial interval of Δ$z_{1}$ = ± 20 μm around the best axial focusing distance. The results are illustrativelly included in Figure 6b at 10 μm steps and, as one can see, we found the maximum tolerable defocusing range to still saving G8-E3 resolved is Δ$z_{1}$ = ± 10 μm. This fact can be seen in both the propagated images and the intensity profiles included through Figure 6b. According to the experimental results achieved for the lateral resolution in Section 3.1.3 (Figure 5c), this is the last element to be resolved at such *z*_1_. Hence, the axial resolution for this *z*_1_ is ρ_axial_ = 20 ± 2 μm. Following this procedure for each *z*_1_, we can obtain the full set of experimental *ρ*_axial_ values, and the results are depicted in Figure 6c. 

These values are then compared with the estimated values when considering Equation (8). In this case, the *NA* considered in this equation is an effective NA, which is computed from Equation (5) considering the lateral resolution values achieved in Section 3.1.3. Figure 6c includes a blue line with these estimated axial resolutions. We can see in Figure 6c that both experimental and estimated values are in good agreement each other, and both of them define a trend of a lost of axial resolution when increasing *z*_1_.

#### 3.1.5. Extended Depth of Field

A major advantage of holographic microscopy in comparison to conventional microscopy is the obtention of an extended DOF. In a compound microscope, the DOF decreases quickly when improving resolution limit, requiring mechanical refocusing to visualize/analyze a large volume. On the contrary, in holographic microscopy, once a digital hologram has been recorded, one can visualize/analyze different objects located at different axial planes by digitally refocusing the recorded optical field, so that three-dimensional (3D) information can be extracted from a two-dimensional (2D) recording/digital hologram [27].

**Figure 7:**
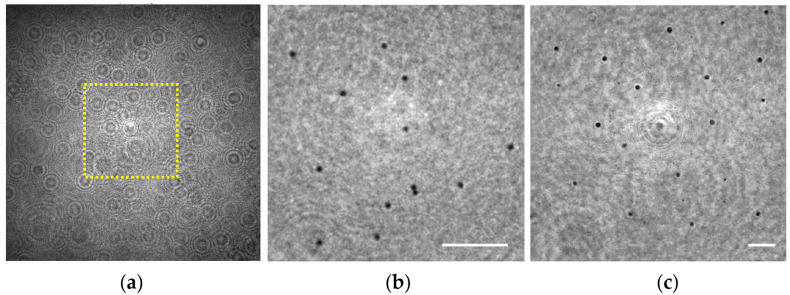
Extended depth of field validation involving 3 μm spheres distributed in two separated 1 mm planes. (**a**) Digital in-line hologram; (**b**,**c**) Retrieved intensity images at the sample planes *z*_1_ = 0.7 ± 0.1 mm and *z*_1_ = 1.7 ± 0.1 mm, respectively. Scale bars in (**b**,**c**) represent 50 μm.

In order to validate this capability for our lensless microscope, we have prepared a sample consisting of two layers of 3 μm diameter beads that are axially separated at two different planes (see Section 2.3). Under these conditions, the hologram recorded by the digital sensor (see Figure 7a) is numerically propagated to both chamber’s sections where the spheres were attached yielding in Figure 7b,c and corresponding with *z*_1_ = 0.7 ± 0.1 mm and 1.7 ± 0.1 mm, respectively, for the region enclosed in the yellow rectangle included in Figure 7a. 

Looking at Figure 7b,c, one can notice that the microspheres placed at both planes can be perfectly visualized and individually identified, thus demonstrating such a DOF expansion of at least 1 mm in depth. In principle, there is no limitation in *z*_1_ distance to be retrieved as long as the weakly diffractive assumption and the requirements of magnification and resolution are fulfilled. Another aspect clearly visible in those reconstructed images is the variation of the lateral magnification. The microspheres located at *z*_1_ = 0.7 mm are magnified with a 21.5X, whereas a magnification of 8.8X is considered for the ones placed at *z*_1_ = 1.7 mm, according to the calibration curve provided in Section 3.1.1 for such *z*_1_. 

#### 3.1.6. Phase Imaging

**Figure 8:**
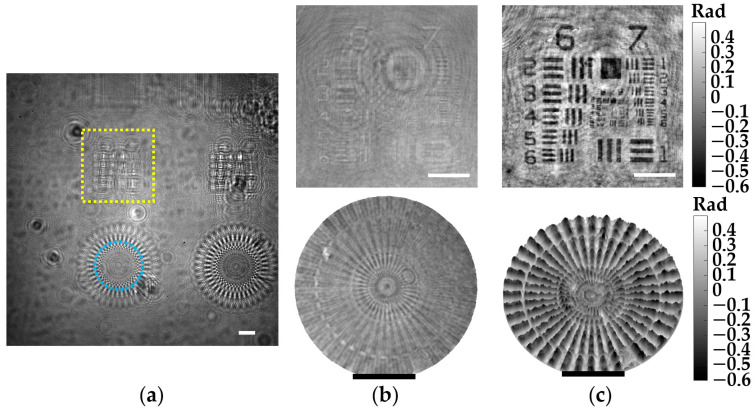
Phase imaging validation of the lensless microscope involving a phase test target. (**a**) Digital in-line hologram for *z*_1_ = 2.2 ± 0.1 mm. (**b**) Retrieved intensity images of the regions marked in (**a**). (**c**) Retrieved 2D (top) and 3D (bottom) phase images of such regions. White and black scale bars represent 100 μm.

The demonstration of phase imaging with our microscope is performed involving a phase test target. Such a phase target is made by Benchmark Technologies and includes several structures with different thickness raised on a Corning Eagle XG glass substrate, having a RI ~ 1.52 for the wavelength of the microscope. Moreover, the thickness of the different elements is perfectly established by the manufacturer, so it is perfect for phase imaging validation. Figure 8 includes the results for two different phase structures located at a *z*_1_ = 2.2 ± 0.1 mm, and has a nominal thickness of 50 nm according to the manufacturer. Here, the recorded in-line hologram is presented in Figure 8a. In addition, Figure 8b shows the intensity distributions of the regions marked with the dashed yellow rectangle and blue circle, enclosing an USAF target and a focus star target, respectively. Furthermore, the retrieved 2D and 3D phase images of these structures are included at the top and bottom of Figure 8c. Since such structures are essentially transparent, they only induce phase delays in the wavefront of the optical field. Hence, they do not present a good contrast in the intensity images, as we can see in Figure 8b. By contrast, these structures can be identified in the phase images (see Figure 8c). It is also worth emphasizing that these structures can be perfectly seen even though they are extremely thin (only 50 nm in thickness).

Regarding the quantitative phase values measured for such structures, these values will be directly affected by the phase disturbances induced by the twin image presence, modifying their values accordingly. However, we can do a rough comparison between the experimental values obtained in Figure 8c and the theoretical values. According to Equation (9), the 50 nm structures should induce a phase delay of 0.4 rad, which approximately matches with the phase values of the color bar in Figure 8c, considering that the background average of the phase images is set to be 0 rad. 

### 3.2. Application to Static Biosamples Inspection

**Figure 9:**
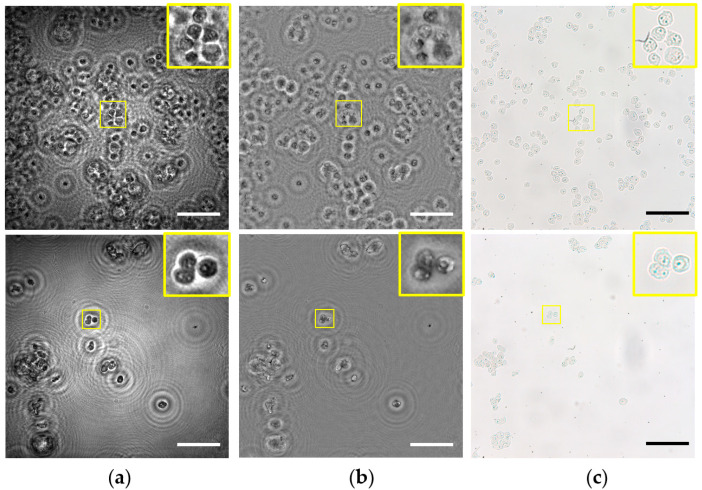
Inspection of different static and unlabeled biosamples. Rows 1–2 include the images for PC-3 and LnCaP prostate cancer cell lines, respectively. (**a**) Intensity and (**b**) phase images retrieved using the proposed lensless microscope. (**c**) 20× intensity images recorded using a bright field microscope. Scale bars are 100 μm.

Next, the lensless microscope is utilized for imaging several fixed and undyed biosamples. More concretely, the biosamples are different types of prostate cancer cells (PC-3 and LnCaP cell lines). The cells are prepared as described in Section 2.3. Figure 9 shows the images provided by the lensless microscope as well as the images recorded using a standard compound microscope (Olympus BX60) with a 20X/0.46*NA* microscope objective for comparison. Thus, intensity and phase images retrieved by our lensless microscope are included in Figure 9a,b, whereas intensity recorded images with bright field microscopy are shown in Figure 9c. 

Looking at the images provided by the lensless microscope, we can visualize the different cellular types. Moreover, magnified areas (yellow squares) including only a few cells serve to identify internal structures, such as nucleus inside the cells. On the other hand, images recorded by the optical compound microscope reveal that such internal structures are indeed present in the cells, and they are not artifacts caused by the twin image or other noise sources. That means our holographic microscope can also image some inner components of the cells, such as the compound microscope using a medium *NA* microscope lens, but with the advantage of having not only intensity but also phase information, which is crucial in biosamples for label-free high-contrast imaging.

### 3.3. Application to Dynamic Biosamples

**Figure 10:**
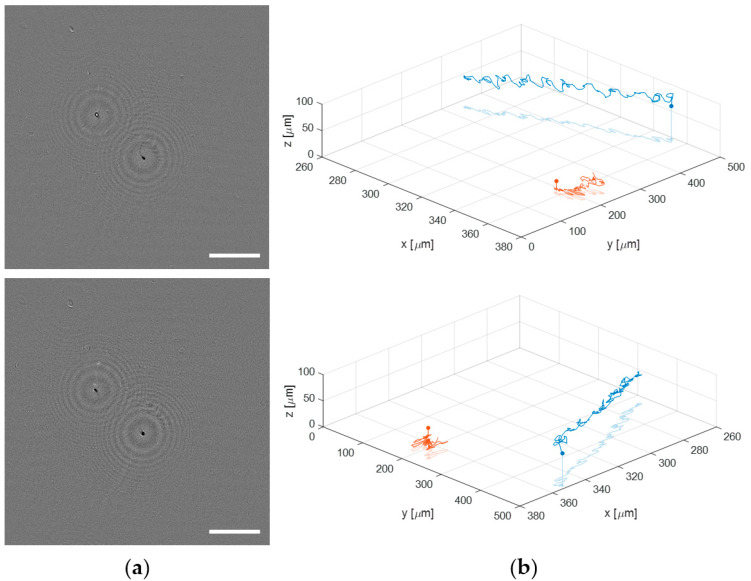
Application of the lensless microscope to the study of the 3D movement of live sperm cells. (**a**) phase contrast images in different planes (Videos S1 and S2, respectively); (**b**) two different perspectives of the final 3D spermatozoid trajectories (Videos S3 and S4).

Finally, the proposed lensless microscope is employed to visualize and track a live human sperm sample. Such a biosample is prepared as described in Section 2.3. When a sperm sample is introduced into a 100 μm counting chamber, the sperm cells are usually flowing through different depths. This fact makes impossible the analysis of the sperm trajectories with conventional microscopy because the limited DOF provided by the microscope lenses. By contrast, the extended DOF of our holographic lensless microscope enables four-dimensional (4D) analysis of flowing sperm cells. Figure 10 shows just an example of the capability of the microscope to obtain the 3D trajectories of sperm cells flowing along different depths (axially separated around 60 μm). Figure 10a include the first frames of a sequence of phase contrast images generated in two different planes, which are separately included in movies Video S1 (top image) and Video S2 (bottom image) (see Supplementary Material). The total recording time is 3 s at a video rate of 100 fps. However, those movies are displayed at 30 fps to slowly see in detail the movement of the sperm cells. In the top image of Figure 10a, the bottom right sperm cell is brought into focus, whereas the top left appears focused in the bottom part of Figure 9a. Additionally, we show the full 3D trajectories followed by the two sperm cells during the recording time in two different perspectives (see Figure 10b), where we include both the 3D movement and the projection on the XY plane of the sperm cells in movies Videos S3 and S4. For obtaining the trajectories, we apply a local focusing criteria based on the recently published DarkFocus numerical autofocusing method [45], but other methods can be also employed instead [46,47].

## 4. Discussion and Conclusions

The presented lensless holographic microscope includes all optical, mechanical, and electronic components in a small quasi-cubic structure (20 × 16 × 13 cm), being easily transportable in a backpack or a bag. In addition, such a microscope is able to obtain images with a lateral resolution up to 1.65 μm (*ρ*_theo_~1 μm, *ρ*_exp_~1.65 μm) in real time and without the use of any lens for imaging. Furthermore, the lateral magnification and *FOV* can be modified by simply changing the axial position of the sample. Moreover, two major advantages of the lensless holographic microscope in comparison to conventional compound microscopes are the extended DOF and phase imaging capability. On one hand, the DOF is usually small (~μm) in a compound microscope and decreases sharply with improving resolution, so that the visualization of a large volume requires of refocusing to different planes. By contrast, in DIHM, a single 2D hologram can be numerically propagated to different planes in order to produce a 3D image of the whole sample volume [26], which has been experimentally validated in Section 3.1.5 by imaging microspheres axially separated 1 mm and exploited in Section 3.3 for the obtention of 3D trajectories of sperm cells. On the other hand, phase imaging is provided very fast by numerically propagating the hologram (in our case, following an ASM) to the object plane, which cannot be achieved with conventional microscopy. That is a great advantage when working with transparent samples, which do not absorb but delay the light, so that phase images normally provide more useful information than intensity images. That is the case of most of the microscopic biosamples, including cells and thin tissue sections. As a major limitation of the microscope, the definition of a Gabor configuration, where the more reference region the more efficacy in the reconstruction process, restricts its use to weakly diffractive samples and can compromise the extended DOF for dense samples. Another negative aspect is the presence of the defocused twin image that limits the quality of the reconstructions. Nonetheless, several methods to reduce or minimize the contribution of such a twin image were reported in the literature [48,49,50,51,52]. Finally, the use of violet light may present two limitations in practical applications. First, digital cameras usually have poor quantum efficiency at 405 nm. Second, it may cause photo damage to the living biological samples, which may induce abnormal responses in dynamic cellular processes under analysis. By contrast, the use of a longer wavelength, such as green light, not only presents a high quantum efficiency, but also is suitable for biological imaging due to biosafety. However, it reduces the system performance in terms of spatial resolution, which may be critical when identification and analysis of internal cellular structures is required.

When comparing our microscope with the existing LHM technologies, it compares favorably in several aspects. For instance, due to its optimized DIHM configuration and optical components, our system experimentally demonstrates higher lateral resolution than most of the reported LHMs [5,14,18,20,21,22], achieving experimental gain factors of around 2 [5,14,18] or even more [20,21,22]. Only a few contributions reported similar lateral resolutions [12,15]. However, those microscopes employ complicated software for imaging [12,15], and in the case of [12], also delicate hardware for illumination, consisting of a LED array and multi-mode fibers. Regarding the *FOV*, our microscope provides bigger *FOV* than other microscopes based on DIHM for the same magnification value [5,14,15,18,21,22], owing to the larger size of the sensor area. Nevertheless, the lensfree on-chip microscopes [7,8,12] present the biggest *FOV*s (around several cm^2^) since they work with a magnification equal to 1. By contrast, although our microscope is compact, portable, and cost-effective, there exist other technologies that are smaller [7,8,12,15,21,22], lighter [7,8,12,21], and cheaper [18,21] than our LHM. On one hand, some LHMs with smaller dimensions are proposed in [15,21,22], whose external dimensions are 9.0 × 8.5 × 17.5 cm^3^, 14.9 × 11.2 × 12.2 cm^3^ and 7.0 × 6.5 × 6.5 cm^3^, respectively, or proposed by Ozcan’s group in [7,8], whose lensfree on-chip microscope presented dimensions of 4.2 × 4.2 × 5.8 cm^3^. On the other hand, although our microscope is very light (around 500 g), there exist lighter LHMs, having a weight of less than 280 g [21], or less than 150 [7,8,12]. Finally, probably the cheapest 3D-printed LHMs were reported in [18,21], whose prices were less than $190 and $52.82, respectively.

Another crucial aspect to consider here is the thorough analysis performed of the principal optical parameters of our microscope. It is worth noting that most of the previous contributions reporting on LHMs [5,7,8,12,14,15,17,18,20,22] only validated their performance for a specific optical configuration, that is, for a given magnification, *FOV*, lateral resolution, etc., without exploiting the versality of lensless configurations to easily provide either higher magnification and resolutions or bigger *FOV*s. In addition, some of those contributions were mainly focused on the biological applicability of such LHMs, rather than to provide an in-depth characterization of the optical parameters as a function of the axial position of the sample. Some of these applications were the analysis of cells [17,18,20], the detection of waterborne parasites [8], or the imaging and tracking of bacteria [14], just to cite a few. In the case of lensfree on-chip microscopes, the characterization of the optical performance as a function of the sample’s axial distance is not as crucial as in the case of LHM based on DIHM configurations, owing to the optical parameters remain practically unchanged. However, in DIHM, such parameters are rapidly altered with the axial distance, and thus a thorough analysis becomes of vital importance. Despite this, only few contributions reporting on DIHM microscopes [6,21] partially provided a more detailed characterization of such an issue. In [6], the authors only estimated the theoretical dependences of the lateral and axial resolutions and *FOV* as a function of the distance, but they did not provide neither experimental validation of them nor comparison between theoretical and experimental values. On the other hand, in [21], the authors provided the theoretical limit values of the range of lateral resolutions and *FOV*s achievable by their LHM, and experimentally demonstrated those parameters for only one (lateral resolution) or three axial positions (*FOV*). By contrast, in our contribution, we have conducted a much more detailed and rigorous analysis, not only of those parameters, but also of magnification and axial resolution, providing theoretical and experimental values and comparing them for several distances, covering a broad range of axial positions.

Hence, in this contribution we have presented a complete analysis of a robust, cost-effective, and high-resolution DIHM for biomedical applications. Several major features such as lateral magnification, *FOV*, and lateral and axial resolutions have been analyzed into detail for different axial positions of the sample by comparing experimental results with the theoretical predictions. Such a lensless microscope has been finally applied for bioimaging involving different types of not only static, but also dynamic, biosamples. The whole analysis performed here may serve as a guide for future developments of other lensless holographic microscopes based on DIHM principle. Due to its reduced size and price, this microscope appears as a great tool to be used in laboratories with limited sources and/or in remote areas, which has applications in sperm assessment, microfluidics, or point-of-care diagnosis, among others.

## Data Availability

Data available on request due to privacy restrictions.

## Supplementary Materials

The following supporting information can be downloaded at: www.mdpi.com/1424-8220/22/2/553/s1, Video S1: Phase contrast movie in one focused plane; Video S2: Phase contrast movie in another focused plane; Video S3: A perspective movie of the 3D sperm tracks; Video S4: An orthogonal perspective movie of the 3D sperm tracks.

## References

[1] Zhu H., Isikman S.O., Mudanyali O., Greenbaum A., Ozcan A. (2013). Optical imaging techniques for point-of-care diagnostics. Lab Chip.

[2] Greenbaum A., Luo W., Su T.-W., Göröcs Z., Xue L., Isikman S.O., Coskun A., Mudanyali O., Ozcan A. (2012). Imaging without lenses: Achievements and remaining challenges of wide-field on-chip microscopy. Nat. Methods.

[3] Roy M., Seo D., Oh S., Yang J.W., Seo S. (2017). A review of recent progress in lens-free imaging and sensing. Biosens. Bioelectron..

[4] Wu Y., Ozcan A. (2018). Lensless digital holographic microscopy and its applications in biomedicine and environmental monitoring. Methods.

[5] Repetto L., Piano E., Pontiggia C. (2004). Lensless digital holographic microscope with light-emitting diode illumination. Opt. Lett..

[6] Jericho S.K., Garcia-Sucerquia J., Xu W., Jericho M.H., Kreuzer H.J. (2006). Submersible digital in-line holographic microscope. Rev. Sci. Instrum..

[7] Mudanyali O., Tseng D., Oh C., Isikman S.O., Sencan I., Bishara W., Oztoprak C., Seo S., Khademhosseini B., Ozcan A. (2010). Compact, light-weight and cost-effective microscope based on lensless incoherent holography for telemedicine applications. Lab Chip.

[8] Mudanyali O., Oztoprak C., Tseng D., Erlinger A., Ozcan A. (2010). Detection of waterborne parasites using field-portable and cost-effective lensfree microscopy. Lab Chip.

[9] Isikman S.O., Bishara W., Sikora U., Yaglidere O., Yeah J., Ozcan A. (2011). Field-portable lensfree tomographic microscope. Lab Chip.

[10] Kim S.B., Bae H., Cha J.M., Moon S.J., Dokmeci R.D., Cropek D.M., Khademhosseini A. (2011). A cell-based biosensor for real-time detection of cardiotoxicity using lensfree imaging. Lab Chip.

[11] Lim Y., Lee S.-Y., Lee B. (2011). Transflective digital holographic microscopy and its use for probing plasmonic light beaming. Opt. Express.

[12] Greenbaum A., Akbari N., Feizi A., Luo W., Ozcan A. (2013). Field-Portable Pixel Super-Resolution Colour Microscope. PLoS ONE.

[13] Pushkarsky I., Liu Y., Weaver W.M., Su T.-W., Mudanyali O., Ozcan A., Di Carlo D. (2014). Automated single-cell motility analysis on a chip using lensfree microscopy. Sci. Rep..

[14] Serabyn E., Liewer K., Lindensmith C., Wallace K., Nadeau J. (2016). Compact, lensless digital holographic microscope for remote microbiology. Opt. Express.

[15] Sanz M., Picazo-Bueno J.Á., Granero L., Garciá J., Micó V. (2017). Compact, cost-effective and field-portable microscope prototype based on MISHELF microscopy. Sci. Rep..

[16] Eom J., Moon S. (2018). Three-Dimensional High-Resolution Digital Inline Hologram Reconstruction with a Volumetric Deconvolution Method. Sensors.

[17] Seo D., Oh S., Lee M., Hwang Y., Seo S. (2018). A Field-Portable Cell Analyzer without a Microscope and Reagents. Sensors.

[18] Amann S., Witzleben M., Breuer S. (2019). 3D-printable portable open-source platform for low-cost lens-less holographic cellular imaging. Sci. Rep..

[19] Brunnhofer G., Bergmann A., Klug A., Kraft M. (2019). Design and Validation of a Holographic Particle Counter. Sensors.

[20] Scholz G., Mariana S., Dharmawan A.B., Syamsu I., Hörmann P., Reuse C., Hartmann J., Hiller K., Prades J.D., Wasisto H.S. (2019). Continuous Live-Cell Culture Imaging and Single-Cell Tracking by Computational Lensfree LED Microscopy. Sensors.

[21] Tobon-Maya H., Zapata-Valencia S., Zora-Guzmán E., Buitrago-Duque C., Garcia-Sucerquia J. (2021). Open-source, cost-effective, portable, 3D-printed digital lensless holographic microscope. Appl. Opt..

[22] Huang X., Li Y., Xu X., Wang R., Yao J., Han W., Wei M., Chen J., Xuan W., Sun L. (2021). High-precision lensless microscope on a chip based on in-line holographic imaging. Sensors.

[23] Gabor D. (1948). A new microscopic principle. Nature.

[24] Rogers G.L. (1952). XIV—Experiments in diffraction microscopy. Proc. R. Soc. Edinburgh Sect. A Math. Phys. Sci..

[25] Xu W., Jericho M.H., Meinertzhagen I.A., Kreuzer H.J. (2001). Digital in-line holography for biological applications. Proc. Natl. Acad. Sci. USA.

[26] Ferraro P., Wax A., Zalevsky Z. (2011). Coherent Light Microscopy: Imaging and Quantitative Phase Analysis.

[27] Garcia-sucerquia J., Xu W., Jericho S.K., Klages P., Jericho M.H., Kreuzer H.J. (2006). Digital in-line holographic microscopy. Appl. Opt..

[28] Frentz Z., Kuehn S., Hekstra D., Leibler S. (2010). Microbial population dynamics by digital in-line holographic microscopy. Rev. Sci. Instrum..

[29] Greenbaum A., Ozcan A. (2012). Maskless imaging of dense samples using pixel super-resolution based multi-height lensfree on-chip microscopy. Opt. Express.

[30] Mendoza-Yero O., Calabuig A., Tajahuerce E., Lancis J., Andrés P., Garcia-Sucerquia J. (2013). Femtosecond digital lensless holographic microscopy to image biological samples. Opt. Lett..

[31] Perucho B., Micó V. (2014). Wavefront holoscopy: Application of digital in-line holography for the inspection of engraved marks in progressive addition lenses. J. Biomed. Opt..

[32] Sanz M., Trusiak M., García J., Micó V. (2020). Variable zoom digital in-line holographic microscopy. Opt Lasers Eng..

[33] Rong L., Latychevskaia T., Chen C., Wang D., Yu Z., Zhou X., Li Z., Huang H., Wang Y., Zhou Z. (2015). Terahertz in-line digital holography of human hepatocellular carcinoma tissue. Sci. Rep..

[34] Garcia-Sucerquia J., Alvarez-Palacio D.C., Jericho M.H., Kreuzer H.J. (2006). Comment on “Reconstruction algorithm for high-numerical-aperture holograms with diffraction-limited resolution”. Opt. Lett..

[35] Kanka M., Riesenberg R., Kreuzer H.J. (2009). Reconstruction of high-resolution holographic microscopic images. Opt. Lett..

[36] Micó V., Zalevsky Z. (2010). Superresolved digital in-line holographic microscopy for high-resolution lensless biological imaging. J. Biomed. Opt..

[37] Kanka M., Riesenberg R., Petruck P., Graulig C. (2011). High resolution (*NA* = 0.8) in lensless in-line holographic microscopy with glass sample carriers. Opt. Lett..

[38] Bishara W., Su T.-W., Coskun A.F., Ozcan A. (2010). Lensfree on-chip microscopy over a wide field-of-view using pixel super-resolution. Opt. Express.

[39] Bishara W., Sikora U., Mudanyali O., Su T.-W., Yaglidere O., Luckhart S., Ozcan A. (2011). Holographic pixel super-resolution in portable lensless on-chip microscopy using a fiber-optic array. Lab Chip.

[40] Lee M., Yaglidere O., Ozcan A. (2011). Field-portable reflection and transmission microscopy based on lensless holography. Biomed. Opt. Express.

[41] Kim M.K. (2011). Digital Holographic Microscopy. Principles, Techniques, and Applications.

[42] Agbana T.E., Gong H., Amoah A.S., Bezzubik V., Verhaegen M., Vdovin G. (2017). Aliasing, coherence, and resolution in a lensless holographic microscope. Opt. Lett..

[43] Latychevskaia T. (2019). Lateral and axial resolution criteria in incoherent and coherent optics and holography, near-and far-field regimes. Appl. Opt..

[44] Goodman J.W. (2005). Introduction to Fourier Optics.

[45] Trusiak M., Picazo-Bueno J.A., Zdankowski P., Micó V. (2020). DarkFocus: Numerical autofocusing in digital in-line holographic microscopy using variance of computational dark-field gradient. Opt. Lasers Eng..

[46] Su T.W., Xue L., Ozcan A. (2012). High-throughput lensfree 3D tracking of human sperms reveals rare statistics of helical trajectories. Proc. Natl. Acad. Sci. USA.

[47] Di Caprio G., El Mallahi A., Ferraro P., Dale R., Coppola G., Dale B., Coppola G., Dubois F. (2014). 4D tracking of clinical seminal samples for quantitative characterization of motility parameters. Biomed. Opt. Express.

[48] Gerchberg R.W., Saxton W.O. (1972). A practical algorithm for the determination of phase from image and diffraction plane pictures. Optik.

[49] Latychevskaia T., Fink H.W. (2007). Solution to the twin image problem in holography. Phys. Rev. Lett..

[50] Latychevskaia T., Fink H.W. (2015). Reconstruction of purely absorbing, absorbing and phase-shifting, and strong phase-shifting objects from their single-shot in-line holograms. Appl. Opt..

[51] Sanz M., Picazo-Bueno J.A., García J., Micó V. (2015). Improved quantitative phase imaging in lensless microscopy by single-shot multi-wavelength illumination using a fast convergence algorithm. Opt. Express.

[52] Latychevskaia T. (2019). Iterative phase retrieval for digital holography: Tutorial. J. Opt. Soc. Am. A.

